# Acquisition of Pneumococci Specific Effector and Regulatory Cd4^+^ T Cells Localising within Human Upper Respiratory-Tract Mucosal Lymphoid Tissue

**DOI:** 10.1371/journal.ppat.1002396

**Published:** 2011-12-01

**Authors:** Jeffrey Pido-Lopez, William W. Kwok, Timothy J. Mitchell, Robert S. Heyderman, Neil A. Williams

**Affiliations:** 1 School of Cellular and Molecular Medicine, University of Bristol, Bristol, United Kingdom; 2 Benaroya Research Institute at Virginia Mason, Seattle, Washington, United States of America; 3 Institute of Infection, Immunity and Inflammation, University of Glasgow, Glasgow, United Kingdom; 4 Wellcome Trust Clinical Research Programme, Blantyre, Malawi; The University of Texas Health Science Center at San Antonio, United States of America

## Abstract

The upper respiratory tract mucosa is the location for commensal *Streptococcus* (*S*.) *pneumoniae* colonization and therefore represents a major site of contact between host and bacteria. The CD4^+^ T cell response to pneumococcus is increasingly recognised as an important mediator of immunity that protects against invasive disease, with data suggesting a critical role for Th17 cells in mucosal clearance. By assessing CD4 T cell proliferative responses we demonstrate age-related sequestration of Th1 and Th17 CD4^+^ T cells reactive to pneumococcal protein antigens within mucosal lymphoid tissue. CD25^hi^ T cell depletion and utilisation of pneumococcal specific MHCII tetramers revealed the presence of antigen specific Tregs that utilised CTLA-4 and PDL-1 surface molecules to suppress these responses. The balance between mucosal effector and regulatory CD4^+^ T cell immunity is likely to be critical to pneumococcal commensalism and the prevention of unwanted pathology associated with carriage. However, if dysregulated, such responses may render the host more susceptible to invasive pneumococcal infection and adversely affect the successful implementation of both polysaccharide-conjugate and novel protein-based pneumococcal vaccines.

## Author Summary

The *S. pneumoniae* bacteria is a major cause of disease (e.g. pneumonia and meningitis) particularly affecting infants. In most cases bacteria can colonise the nose without causing harm, however colonisation is thought to be a prerequisite of disease. With increasing age colonization and disease, rates gradually decrease which is likely due to the development of immunity to the pneumococcus with age. The CD4 T cells of the immune system may contribute to the defence against bacterial colonisation by producing factors that promote pneumococcal killing. Herein, we show that CD4 T cells reactive to pneumococci are found in greater numbers at the site of colonisation and gradually increase in their levels from infancy. However, at the peak of CD4 T cell responses from late teens, we detected the presence of regulatory T cells (Tregs) which suppressed anti-pneumococci CD4 T cell activity greatly. Our finding shows that pneumococcal reactive CD4 T cells selectively populate colonisation sites and increase with age as a result of ongoing bacterial exposure throughout life, inversely correlating with colonisation and disease rates. As factors that utilise CD4 T cells become increasingly advocated as potential preventative strategies against pneumococcal carriage and disease, the observed effect of Tregs must be considered.

## Introduction

Global estimates suggest that approximately one and a half million deaths due to pneumonia, bacteraemia and meningitis are associated with pneumococcal infection annually, around two thirds of these occur in children in resource-poor countries [1]–[3]. In addition to this high disease burden, *S. pneumoniae* is also a common commensal of the upper respiratory tract colonising approximately 40–50% of children from 0 to 2 years of age in the United Kingdom [4] and up to 90% of African children in this same age group [5]. It is assumed that this commensal relationship is regulated by natural immunity to the pneumococcus, which is acquired from early infancy onwards [6]. This immunity is thought to result in a gradual decline in pneumococcal carriage and infection with increasing age, even in settings where the rates of invasive pneumococcal disease are high [7], [8].

Classically, due to the undeniable protective efficacy of pneumococcal capsular polysaccharide vaccines, anti-capsular antibodies have been thought to be largely responsible for natural immunity to *S. pneumoniae* [6], [9]. As a consequence, studies assessing T cell immunity to the pneumococcus, particularly in humans, have until recently been lacking. However, re-evaluation of the epidemiology has brought into question the central role of anti-capsular antibody [6]. Studies of colonization, antibody acquisition and the relationship with otitis media suggest that naturally-induced antibodies to pneumococcal protein antigens may be protective against disease [10]. The demonstration of CD4^+^ T cells that respond to pneumococcal protein antigens points to the possible contribution of these cells to the development of serotype independent protection against *S. pneumoniae* and the age-related decline in pneumococcal disease [6], [11]–[15]. Experiments in the mouse have shown cell-mediated immunity to be an important protagonist in host immune defence against pneumococcal colonization following immunization with protein antigens. These studies have implicated the Th17 CD4 T cell subset in the promotion of mucosal clearance through the recruitment of neutrophils and macrophages. Indeed, it has been suggested that pneumolysin (Ply), a cytotoxic protein antigen and TLR4 agonist which elicits protective immune responses in rodent challenge models, is essential to the generation of Th17 responses to *S. pneumoniae* [11], [14], [16].

We have investigated the nature of CD4 T cell immunity in the upper respiratory tract with increasing age, and the relationship between immunity at this site and that seen in the circulation. In addition, we have determined whether pneumococcus-specific Treg cells arise as a result of natural exposure and whether such cells modulate the nature of protective responses.

## Results

### Anti-pneumococcal CD4^+^ T cell responses are greater in mucosal lymphoid tissue than in the blood

**Figure 1:**
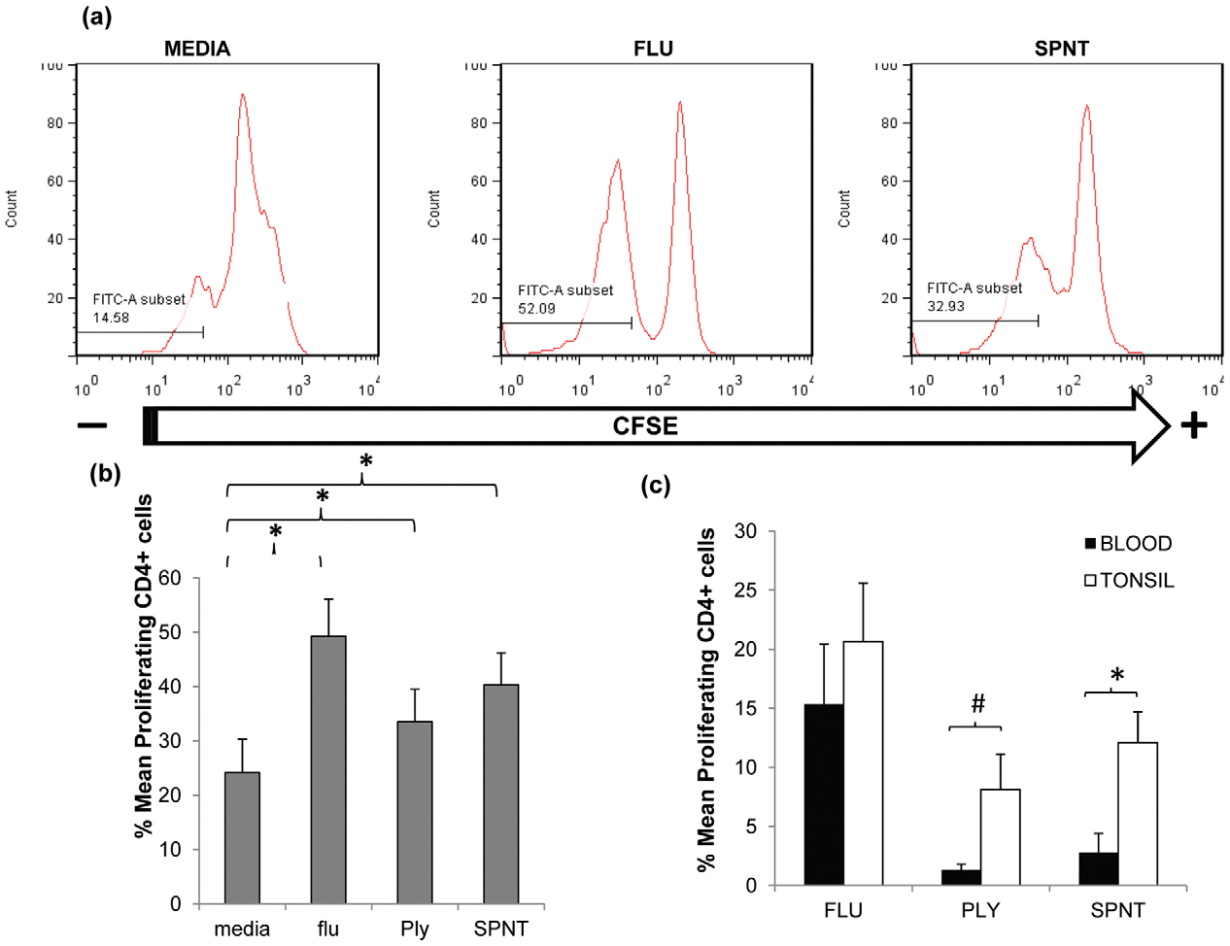
Anti-pneumococcal CD4 T cells proliferative responses in adult tonsils and blood during *in vitro* pneumococcal peptide antigen challenge. (**a**) A typical FACS plot for CFSE staining within CD4^+^ cells post simulation with flu or SPNT compared to unstimulated (media alone) cells. (**b**) Purified tonsil MNCs (*n* = 8) were stimulated over 9 days with flu or recombinant Ply peptides, or D39 bacterial SPNT or media. CD4^+^ cells identified by FACS staining were assessed for their proliferative responses by CFSE staining. Percent of proliferating CD4^ +^ cells post flu, Ply or SPNT stimulation were all significantly higher than media control (*  = *p* <0.05). (**c**) Greater proliferative responses to pneumococcal peptides by tonsil compared to blood CD4^+^ cells. Tonsil MNCs and PBMCs from the same individuals (*n* = 5), were purified and stimulated *in vitro* with flu, Ply or SPNT and CD4^+^ cell proliferation assessed after 9 days. No significant difference was observed between tonsil (open bars) and blood (filled bars) CD4^ +^ responses to flu but were significant to SPNT (*  =  *p* <0.05) and almost significant (#  =  *p* 0.06) for Ply. Values were calculated with the background (i.e. media alone) proliferation subtracted. Error bars show the SEM.

*S. pneumoniae* can inhabit the upper respiratory tract, particularly during childhood [6], [10] leading to nasopharyngeal colonisation [17], [18]. Multiple colonization events are likely to occur throughout life commencing from early infancy when they are most frequent. Due to ongoing bacterial exposure in the upper respiratory tract, lymphoid tissues within this anatomical site are likely locations for immune induction and depots for pneumococcal reactive lymphocytes. Support for this comes from a previous study assessing tonsil and blood CD4^+^ T cell responses to pneumococcal proteins which hinted at higher responses by tonsillar CD4^+^ T cells compared to those from blood [13]. To test this, we first compared CD4^+^ T cell responses to *S. pneumoniae* in upper respiratory tract lymphoid tissue with that in blood. Tonsil mononuclear cells (MNC) were cultured with recombinant pneumococcal Ply mutant protein [19] or supernatants generated from the culture of type 2 D39 *S. pneumoniae* bacterial cells (SPNT) as described previously [10], [12]. CD4^+^ T cell proliferation was assessed after seven to nine days stimulation via 5, 6-Carboxyfluorescein diacetate succinimidyl ester (CFSE) staining and flow cytometric analysis (Figure 1a). When similar data from eight adult subjects (>20years old) was analysed, the mean percentage of CD4+ T cells proliferating to Ply was 33.5% (±6%) and SPNT was 40.3% (±5.9%). Proliferation to a previously established positive control, influenza [20] was 49.2% (±6.9%) and to the negative control of media alone, 24.1% (±6.2%); thus proliferative responses to all three antigens were significantly greater (*p* <0.05) than the background proliferation (Figure 1b). The results reveal that anti-pneumococcal CD4^+^ T cell responses are evident in the palatine tonsil mucosal lymphoid tissues of adults.

In order to compare the anti-pneumococcal immune responses in the tonsil with those systemically, we analysed paired tonsil and peripheral blood mononuclear cells (PBMC) CD4 T cell proliferative responses. Comparison of CD4^+^ cell proliferative response to both Ply and SPNT stimulation similarly revealed stronger anti-pneumococcal responses by tonsil compared to blood CD4^+^ T cells (Figure 1c). This stronger tonsillar response occurred despite the subtraction of a slightly higher level of background response in a number of the tonsil samples, which might have been expected to mitigate against seeing such a difference. The response to influenza was similar in both compartments. These results suggest that pneumococcal-specific CD4^+^ T cell may be preferentially sequestered within mucosal lymphoid tissues located in the upper respiratory tract, the anatomical site for bacterial colonization.

**Figure 2:**
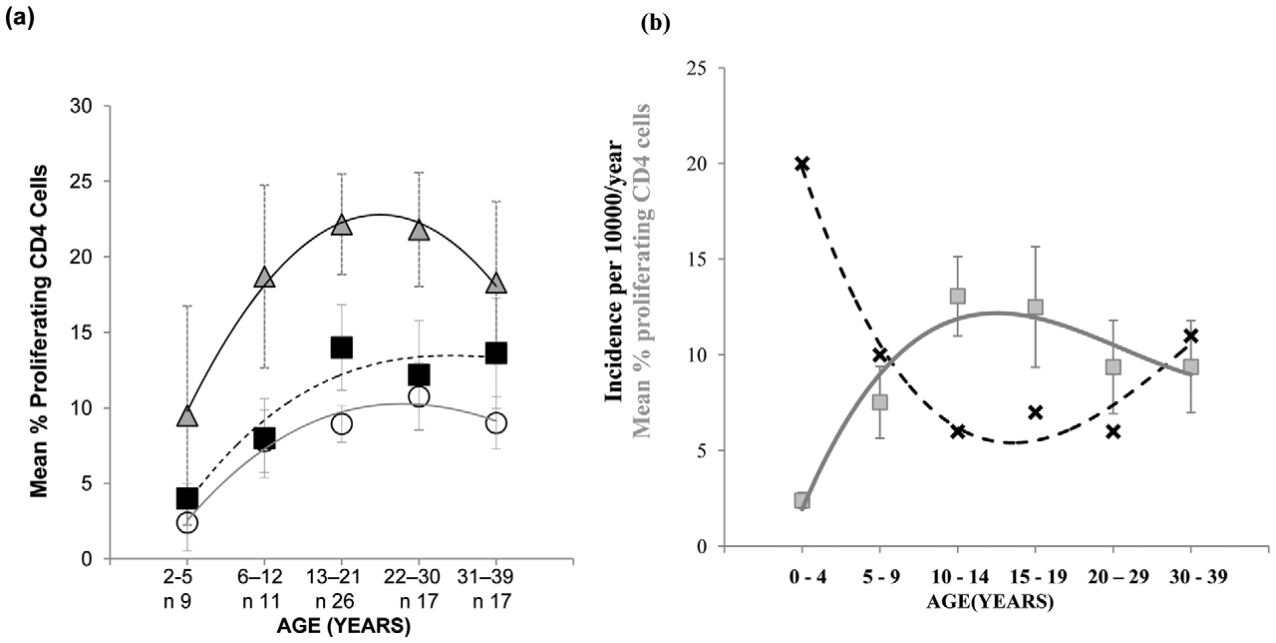
Mucosal CD4 T cell responses to pneumococci during aging and its relation with CAP rates. (**a**) Mucosal CD4 T cell responses to pneumococcal peptide antigen display gradual age-related increases from early childhood until mid-20's and remain relatively constant until mid-life. Tonsil MNCs from subjects (*n* = 80) between the ages of 2 to 39 years and grouped into five age groups were assessed for their CD4 T cell proliferative responses to *Strep. Pneumoniae* Ply (circles, solid grey line of best fit), SPNT (square, dashed black line of best fit) and flu (triangle, black line of best fit) peptides, error bars show SEM. (**b**) Graph for data observed by Myles *et al* showing the trend (black crosses, dashed black line of best fit) of incidence rates of CAP per person/year in the UK at different age groups between 1991-2003 (*n* = 56332, R^2^ = 0.97) in relation to the trend (grey circles, solid grey line of best fit) of mean total (to Ply and to SPNT) anti-pneumococcal CD4 T cell proliferative responses between the ages of 2 to 39 years old (R^2^ = 0.93). Graph for CAP data was generated with permission from P.R. Myles.

As the decline in pneumococcal carriage and invasive disease is thought to be the consequence of age-associated acquisition of natural adaptive anti-pneumococcal immune responses [4], [5], we next examined the mucosal CD4 T cell- mediated response to the pneumococcus from early infancy to mid-life. Evaluation of the proliferative response to Ply and SPNT by CD4^+^ cells of subjects aged between 2 to 39 years old revealed a gradual increase in responses with age, up to around early to mid-twenties and then a plateau thereafter (Figure 2a). For anti-Ply responses the average rate of increase in the percentage of proliferating CD4^+^ cells between ages 2 to 12 years was calculated to be 0.34% per year while for SPNT this was at 0.4% per year. From early teens until 30 years rates dropped to 0.11% proliferating CD4^+^ cells per year for both anti-Ply and anti-SPNT responses. Anti-influenza responses showed a similar trend but at a higher intensity at all the age groups analysed. Interestingly, when we assessed the results of a separate study analysing the age-associated rates of community-acquired pneumonia (CAP) which is primarily due to *S. pneumoniae* infection, in the UK between 2000 to 2003 [21], we observed that the combined proliferative responses of CD4 T cells to SPNT and Ply showed an inverse relationship to the levels of CAP (Figure 2b, generated with permission from P.R. Myles). This finding suggests a potential role for the cell mediated anti-pneumococcal response in protecting from disease.

### Anti-pneumococcal responses by mucosal CD4 cells are regulated by CD25^hi^ Treg cells

**Figure 3:**
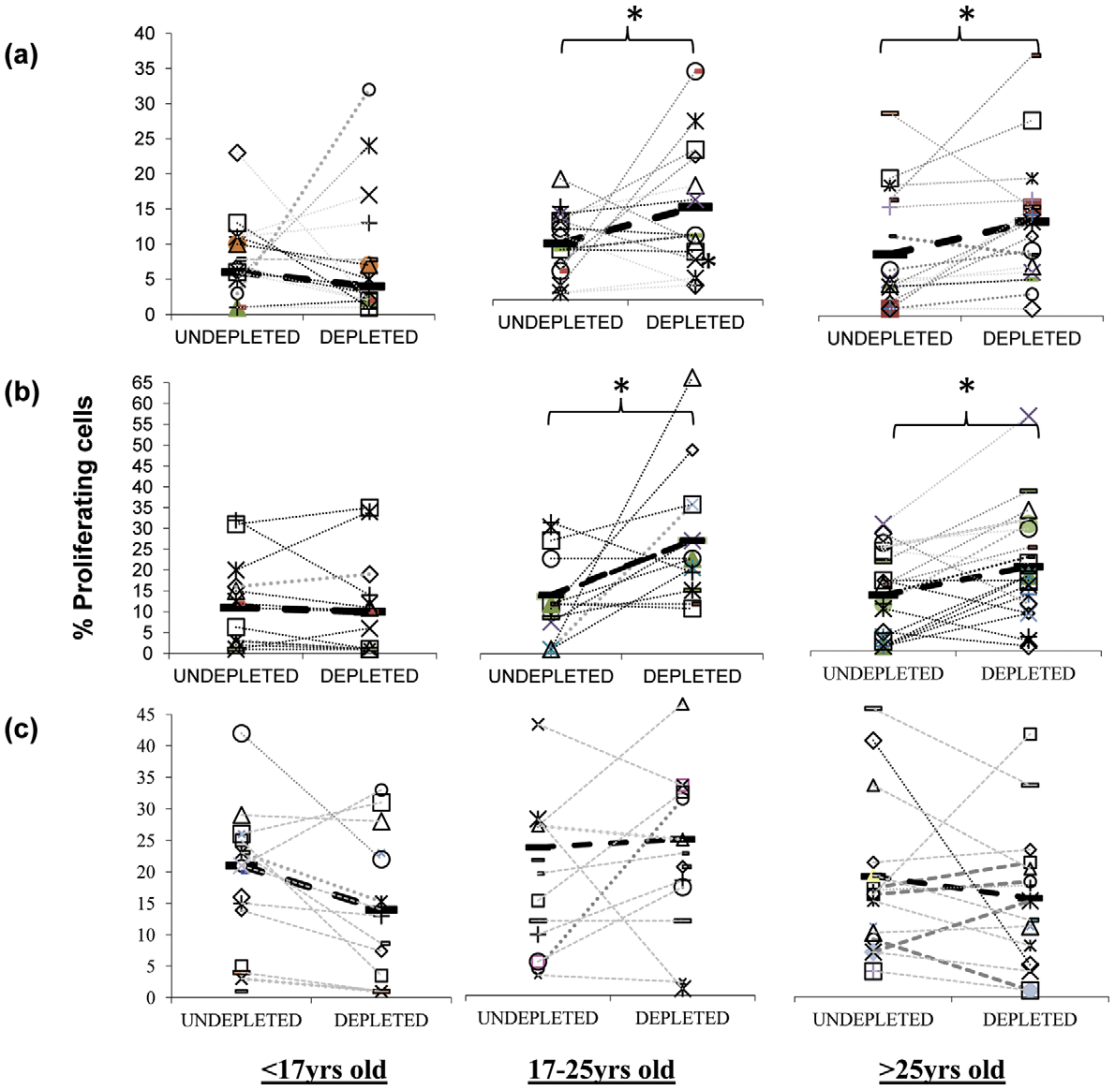
Inhibitory action of anti-pneumococcal responses by regulatory T cells. Inhibition of mucosal CD4^+^ T cell anti-pneumococcal responses to Ply (**a**) and SPNT (**b**) but not to flu (**c**) by CD25^hi^ regulatory T cells in subjects above 16 years old as indicated by increased cell proliferation following depletion of CD25^hi^ cells from tonsil MNC population is observed. Subjects (*n* = 50) were grouped into those aged less than 17 yrs, 17 to 25 yrs and >25 yrs. Individual subject's proliferative response pre and post CD25^hi^ cell depletion are shown with a connecting dashed grey line, while solid black bars and black dashed line represent mean proliferative values for undepleted and CD25^hi^ cell depleted populations. (*  = *p* <0.05).

T regulatory cells are increasingly being recognised as important modulators of responses to bacteria and viruses [22]–[26]. Interestingly, animal studies have revealed that the presence of commensal bacteria can induce Treg levels *in vivo* [27], and similarly, we have established that Treg appear during the acquisition of natural immunity to *Neisseria (N.) meningitidis*, another upper respiratory tract coloniser [20]. Tregs can have unfavourable effects on host immunity by preventing the generation of effective immune responses. For example in humans, elevated Treg numbers are associated with chronic viral infections such as hepatitis B and HIV with the depletion of these cells *in vitro* resulting in improved T cell responses [28], [29]. We therefore assessed the role of Tregs on the mucosal anti-pneumococcal responses by CD4+ T cells [30], [31]. Previous works have shown that many, although not all populations of Tregs can express the IL-2 receptor subunit CD25 at high levels [22]–[25]. We therefore investigated whether pneumococcus-specific Treg cells are present in the tonsils by depleting the Treg cell containing CD25^hi^ cell population from cultures of tonsil mononuclear cells (MNC) in order to assess the impact of such cells on mucosal anti-pneumococcal CD4 T cell responses. We found a significant increase in the proliferative responses of tonsil CD4^+^ T cells to Ply (Figure 3a) and SPNT (Figure 3b) after CD25 depletion in the majority of subjects at the age of 17 years and above but not for those below 17 years. As was observed previously [20], analysis of the CD4 cell anti-influenza responses revealed no significant effect of CD25^hi^ cell depletion on the level of proliferative responses to influenza (Figure 3c).

**Figure 4:**
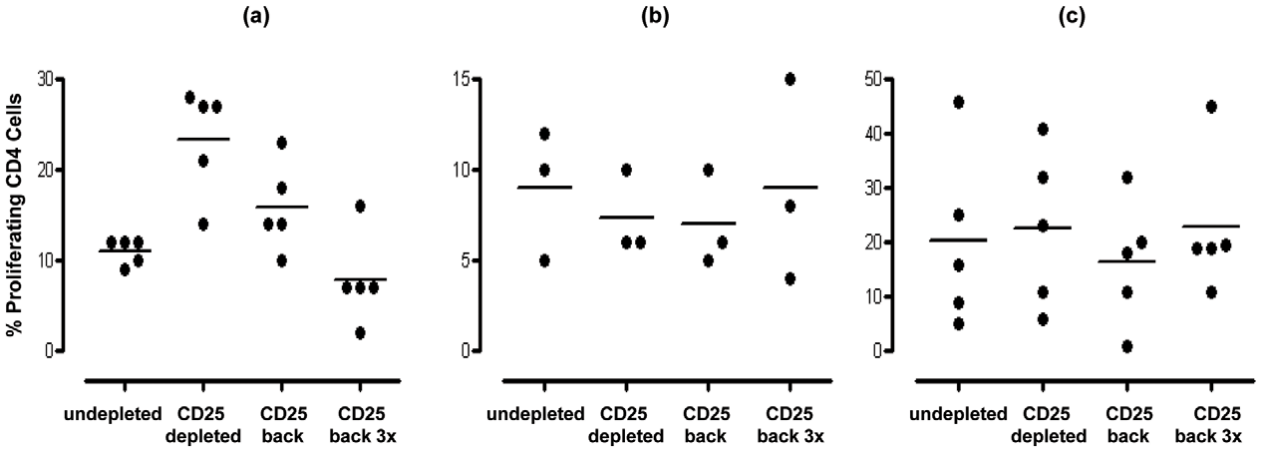
Effect of restoration/addition of Tregs (CD25^hi^) cells back into CD25^hi^ cell depleted MNC samples on proliferative responses by pneumococcal specific CD4^+^ T cells. Tonsil MNCs were depleted of CD25^hi^ cells and left or CD25^hi^ cells added back at the original proportion (∼10%) or at three fold the original proportion (i.e. 30%). Cells were obtained from individuals (**a**) >16years (*n* = 5) and stimulated with SPNT, (**b**) <17 years (*n* = 3) and stimulated with SPNT or individuals (**c**) >16 years and stimulated with flu (*n* = 5). Percentage of proliferating CD4^+^ cells are shown for each individual (open circles) as well as mean (black bars) proliferation in undepleted or CD25^hi^ depleted or CD25^hi^ depleted with CD25^hi^ added back at the original proportion or CD25^hi^ depleted with CD25^hi^ added back at three times the original proportion. (*  = *p* <0.05).

In order to confirm that the enhanced proliferation observed following the depletion of CD25^hi^ cells was as a result of this population exerting an inhibitory effect in undepleted cultures, we subsequently added back the CD25^hi^ cell fraction to the depleted MNC population at the original proportion and at three times the original proportion. Assessment of those subjects (four of five) showing notable increased CD4^+^ T cell proliferation to pneumococcus post Treg depletion revealed that restoration of CD25^hi^ cell numbers resulted in the reversal of the observed increased proliferative responses to Ply (Figure 4a) in subjects above the age of 17 years. When CD25^hi^ cells were added back at three times the original percentage, the proliferative responses decreased even further. When subjects below the age of 17 years were assessed (Figure 4b), no notable changes in the proliferative response were observed between the undepleted, CD25^hi^ depleted, CD25^hi^ added back cell cultures. In keeping with the lack of effect of CD25^hi^ cell depletion on influenza specific CD4^+^ cell responses, no significant change in the proliferative response to influenza were observed following the removal of, or the addition back of this population to depleted MNC populations, even at three times their normal frequency (Figure 4c). Collectively, these data reveal that Treg cells are present within mucosal lymphoid tissues of adults and are able to significantly suppress the proliferative responses of CD4^+^ T cells to *S. pneumoniae*.

### Mucosal T cell immunity to S. pneumoniae is dominated by Th1 and Th17 responses

**Figure 5:**
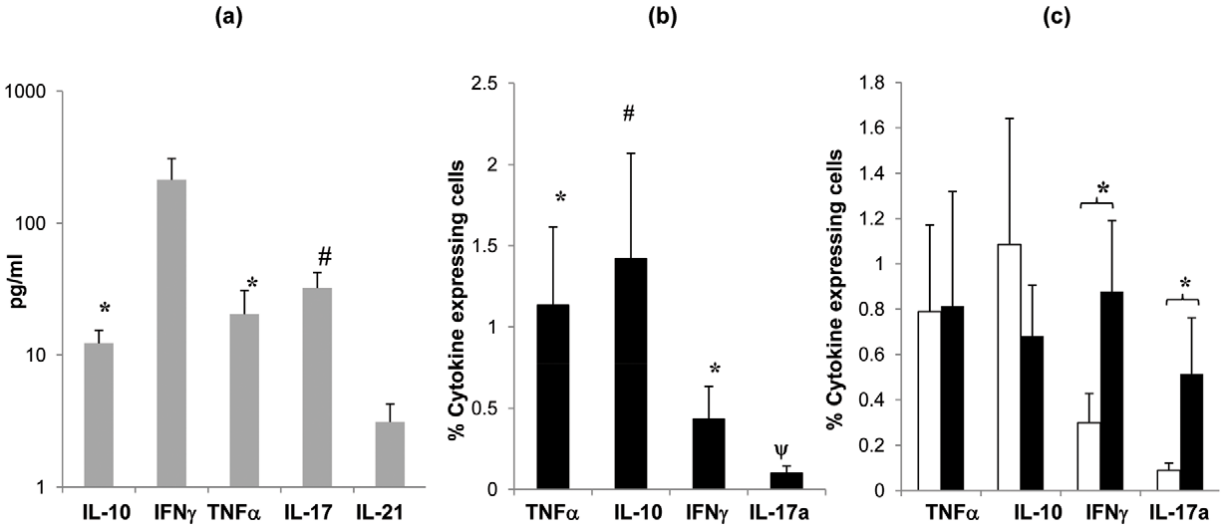
Cytokine profile of tonsil MNCs following *S. pneumoniae* antigen stimulation. Cytokine (IL-10, IFNγ, TNFα, IL-2 and IL-17) production by tonsil MNCs following stimulation with Ply (grey bars, SEM are shown by error bars)) was assessed by quantifying cytokine levels in cell culture supernatants (*n* = 8) at 7 days post cell stimulation by Luminex assay. Mean values are shown for each subject's cytokine levels post Ply stimulation with background cytokine levels in media alone subtracted, individuals with <7% proliferating CD4^+^ cells post Ply stimulation were not assessed (**a**). Cytokine producing CD4^+^ cells were analysed by intracellular cytokine FACS analysis for (IL-10, IFNγ, TNFα and IL-17) to determine cytokine production specifically by CD4^+^ cells post Ply stimulation (*n* = 10). Filled bars show mean percentage of cytokine expressing CD4^+^ cells with the background (media alone) percentage values subtracted (**b**). Cytokine production by CD4^+^ cells post Ply stimulation in undepleted (open bars) and CD25^hi^ cell depleted (filled bars) tonsil MNCs (*n* = 13) was compared by intracellular FACS analysis. Similarly, values represent the mean percent of CD4^+^ cells that are expressing cytokine, with the background (media alone) values subtracted. Only the subjects showing >5% increased proliferation post CD25^hi^ cell depletion were assessed (**c**). All error bars represent SEM (*  = *p* <0.05 and *#*  = *p* 0.06).

CD4 T cells produce and secrete a variety of cytokines that control and co-ordinate effector mechanisms involved in pathogen clearance. In order to determine the nature of the anti-pneumococcal immune response in the mucosa, we assessed cytokine production in supernatants taken from cultures of palatine tonsil MNC following in *vitro* stimulation with Ply. We observed significant production of TNF-α, IL-2 and IL-10 (*p <*0.05) and a modest but not significant increase in IL-17 (*p* = 0.06) compared to the levels observed in unstimulated cultures (Figure 5a). In order to confirm the cellular origin of the pneumococcal antigen-mediated cytokine production, we performed similar studies using intracellular cytokine staining in combination with staining for CD4 receptor. Flow cytometric analysis after 6 to 7 days stimulation and then an overnight re-stimulation with Ply revealed significant increases in CD4 T cell IFN-γ, TNF-α and IL-17 (Figure 5b).

To determine whether the anti-pneumococcal cytokine response is modified by Treg-mediated suppression, we then analysed cytokine production following the removal of the Treg containing CD25^hi^ cell population from the tonsil MNC cultures pre pneumococcal challenge. Intracellular flow cytometric analysis revealed increases in IFN-γ and IL-17 positive cell numbers in the Treg depleted cell cultures. As expected, analysis of those few subjects not displaying increases in anti-pneumococcal CD4 proliferation post Treg depletion revealed little change in their IFN-γ and IL-17 expressing CD4 T cell numbers following CD25^hi^ cell depletion (data not shown). The results indicate an inhibitory effect of Tregs on the pneumococcal-induced production of these specific cytokines by mucosal CD4 T cells. No significant effect on TNF-α and IL-10 production was observed following the depletion of CD25^hi^ cells.

### Cell surface CTLA-4 and PDL-1 molecules are involved in the inhibition of anti-pneumococcal CD4 T cell proliferation by Tregs

**Figure 6:**
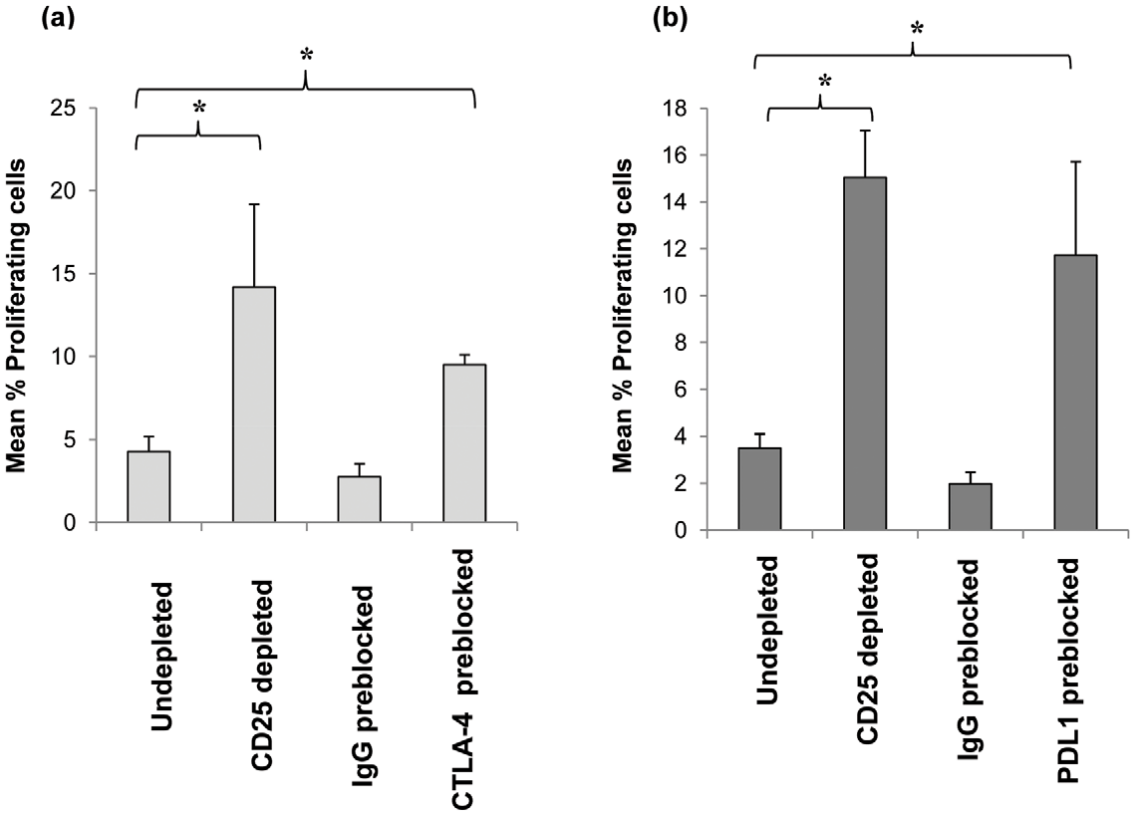
Effect of blockage of CTLA-4 and PDL-1 on CD25^hi^ cells their suppression of anti-pneumococcal proliferative responses by CD4^+^ cells. Purified CD25^hi^ cells were preblocked with anti-human CTLA-4 or anti-human PDL-1 blocking antibodies or isotype control (IgG) antibodies and added back to CD25^hi^ depleted MNCs (i.e. CD25^-^ cells) at the same original proportion and then stimulated with SPNT over 8 days and CD4^+^ cells proliferation analysed. Graph shows the mean percentage of proliferating CD4^+^ cells post SPNT stimulation in the anti-human CTLA-4 (**a**) or anti-human PDL-1 preblocked CD25^hi^ cells (**b**) groups (*n* = 5 each group). (*  = *p* <0.05).

Initial transwell experiments indicated the involvement of cell contact-dependent mechanism/s for the observed Treg suppression of CD4 T cell proliferation (data not shown). We therefore assessed whether Tregs utilised the inhibitory cell surface molecules CTLA-4 and PDL-1 [29], [32]–[37] during their suppression of anti-pneumococcal responses by tonsil CD4^+^ T cells. This was achieved by pre-blocking purified CD25^hi^ Tregs with neutralizing antibodies prior to addition back into CD25^hi^ depleted cell population and subsequent 8 days *in vitro* culture in the presence of SPNT. Flow cytometric analysis revealed that approximately 80% of CD4^+^ CD25^hi^ Tregs were successfully bound by the antibodies while up to 2% of non Tregs cells in the culture became bound by the neutralizing antibodies during the entire 8 day culture period (data not shown) indicating blocking was specific to the Treg cell population. Blocking with CTLA-4 antibody but not an isotype matched IgG1 antibody control resulted in a significant increase in the mean percentage CD4 proliferation from the undepleted culture (Figure 6a). Just as depletion of CD25^hi^ cells resulted in a significant increase (*p* <0.05) in the mean percentage of CD4 T cell proliferation, pre-blocking with PDL-1 but not the isotype matched control IgG2 antibody on CD25^hi^ cells and their subsequent restoration back into CD25^hi^ depleted cell populations also resulted in significant increase (*p* <0.05) in mean CD4 cell proliferation (Figure 6b). Collectively, these data suggests that the suppression of mucosal anti-pneumococcal T cells by Treg involves surface interactions via CTLA-4 and PDL-1 inhibitory co-receptors. Blocking either CTLA-4 or PDL-1 did not increase proliferative responses up to the levels observed in post CD25^+^ cell depletion indicating additional mechanisms may also be involved for Treg suppression of anti-pneumococcal CD4 T cell responses.

### Detection of anti-pneumococcal Ply Treg cells in mucosal tissue

**Figure 7:**
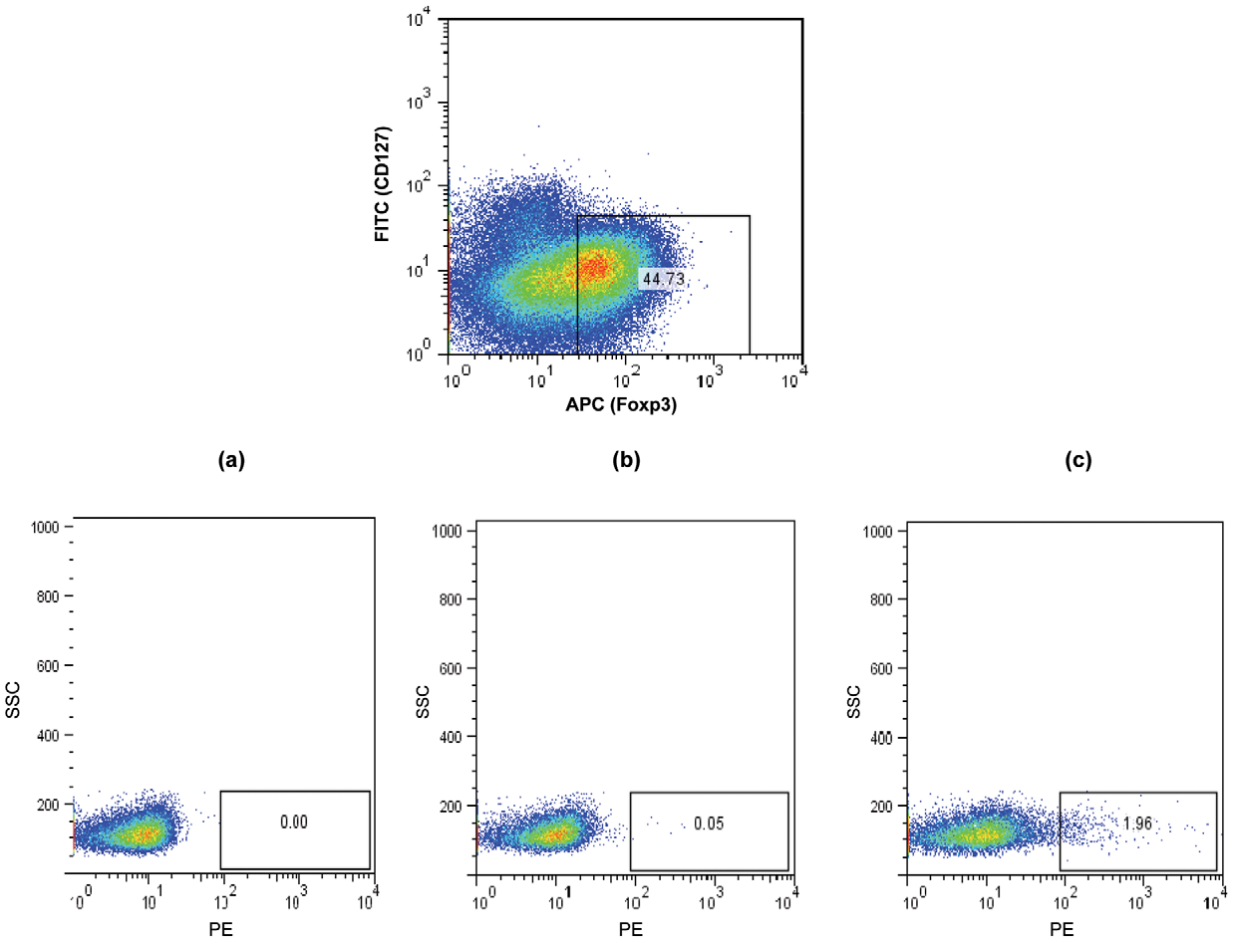
Detection of Ply specific CD127^low/-^ FoxP3^+^ CD4^+^ Treg cells in CD25 enriched tonsil and blood MNC. CD25 enriched MNC were stained with anti- CD127 and anti FoxP3 to allow identification of Tregs and with (**a**) strepavidin-PE, (**b**) negative control Ply tetramer-PE and (**c**) Ply tetramer-PE and analysed by FACS. A typical example of a FACS plot is shown. While the CD127^low/-^ FoxP3^+^ CD4^+^ Treg cells show low level staining at 0.0% with strepavidn-PE (**a**) and negative control Ply-tetramer at 0.05% (**b**), a significantly higher percentage of Treg cells are bound by Ply-tetramers at 1.96%(**c**).

**Figure 8:**
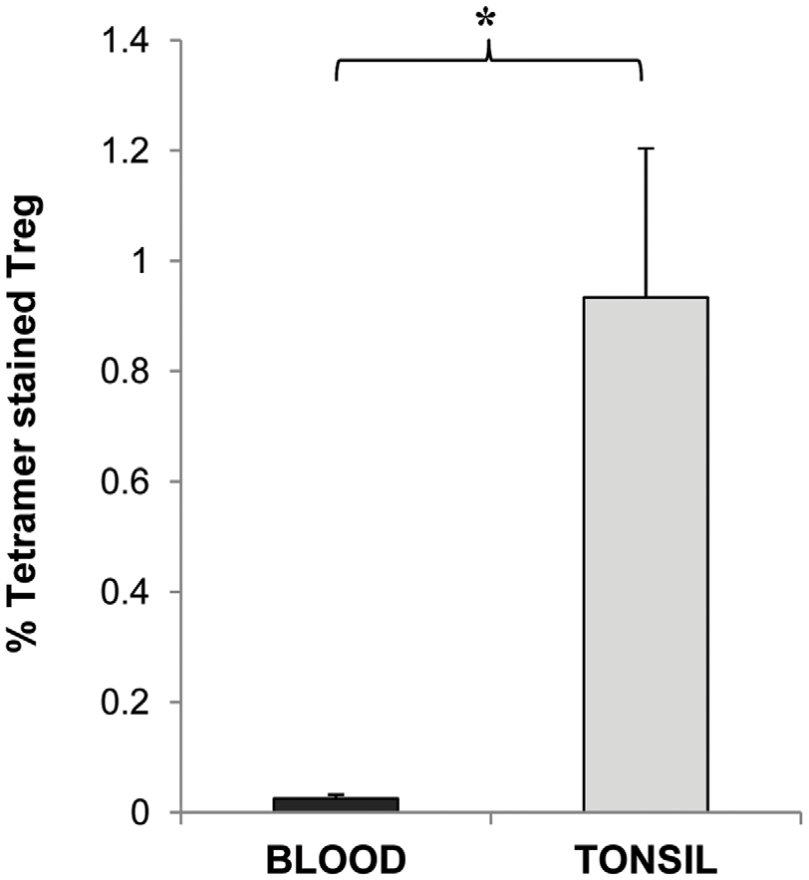
Frequencies of Ply specific Tregs in the tonsil compared to blood. CD127^low/-^ FoxP3^+^ CD4^+^ Treg cells in CD25 enriched blood and tonsil MNC populations were stained with Ply tetramer-PE and assessed by FACS for percentages of Treg cells specific for Ply in the two compartments. Graph shows the mean % of Tregs bound by Ply tetramer in blood (black bars) and in tonsils (grey bars) with % of Tregs stained with control tetramer subtracted (*n* = 3 for blood group and  = 5 for tonsil group).

MHCII tetramers consisting of four identical biotinylated MHCII molecules presenting an epitope of a specific antigen, ligated to one another via fluorescently labelled strepavidin molecules have been increasingly utilised to allow the flow cytometric detection and quantification of CD4 T cells with specificity to the antigen being presented. The target CD4 T cells bind to the antigenic epitope/tetramer complex via their surface T cell receptors (TCR) thus allowing their subsequent detection [38]. We therefore utilised this technology in order to determine whether CD25^+^ Tregs which we observed to inhibit CD4 T cell proliferative responses to Ply can be detected within tonsillar MNC populations. Using Ply epitope presenting MHCII tetramers we confirmed the presence of *S. pneumoniae* specific Treg cells within the tonsil CD25^+^ cell population of adults by generating phycoerythrin (PE)-labelled HLADR04 tetramers bound to one of three different Ply peptide epitopes, or to a non-epitope peptide from Ply as a tetramer-Ply epitope negative control, which was observed to bind poorly to Ply specific cell lines we had generated (data not shown). The HLADR0401 MHCII molecule was chosen as a large fraction of the Caucasian population expressed this particular serotype, thus increasing the chances of finding suitable HLADR0401 subjects for subsequent tetramer analysis. Tetramer binding was assessed on tonsil CD25 enriched cells by flow cytometry (Figure 7). Cells were stained with streptavidin-PE alone (Figure 7a), the tetramer-Ply negative control (Figure 7b), or three tetramer-Ply epitope cocktail (Figure 7c). Approximately 45% of CD25 enriched CD4^+^ cells were found to be FoxP3^+^/CD127 ^low/-^ Treg cells. Within this population 0.01% of cells bound to streptavidin PE alone and 0.47% bound to the control tetramer. Importantly, 1.96% of the Treg bound to the Ply epitope tetramers. In five individuals over 17 years old assessed, 0.53 to 1.96% (with % of cells binding to streptavidin-PE control subtracted) of Treg cells were Ply-specific, which was significantly greater (*p* <0.05) than the 0.005 to 0.46% (with % of cells binding to streptavidin-PE control subtracted) of Treg cells bound by the negative control Ply tetramer. Similar assessment of blood Treg population indicated lower frequencies of Ply specific Treg cells as indicated by a lack of tetramer staining (mean 0.02%) of CD4^+^ FoxP3^+^/CD127^low/-^ peripheral blood mononuclear cells (Figure 8). This result would therefore indicate a similar sequestration of pneumococcal specific Treg cells within the tonsils as that observed for pneumococcal specific conventional CD4 T cells, and also implies that the low CD4 T cell responses in blood is not due to a high frequency of anti-pneumococcal Tregs residing within this compartment and suppressing immunity. Thus using these novel tools we have shown the presence of tonsil FoxP3^+^/CD127^low/-^, CD25 enriched CD4^+^ Treg cells that are specific for pneumococcal Ply antigen, likely contributing to the observed suppression of anti-pneumococcal CD4 T cell responses.

## Discussion

In the current study, the magnitude of CD4 T cell response to *S. pneumoniae* with age was assessed in view of the reported age-related decline in pneumococcal disease and the proposed involvement of cell mediated immunity to this acquisition of protection from disease. We hypothesised that assessment of CD4 T cells residing within upper respiratory tract mucosal lymphoid tissues that are in close proximity to the site of *S. pneumoniae* colonization may prove to be more appropriate for the study. Indeed, comparison of anti-pneumococcal responses by CD4 T cells from blood and palatine tonsils revealed a greater level of responsiveness by the cells obtained from the latter source. Although, it must be acknowledged that tonsils are only available from individuals who have either an upper airway obstruction or have suffered from recurrent tonsillitis, the results are highly suggestive of increased numbers of pneumococcal specific CD4^+^ T cells within lymphoid tissues in the vicinity of the nasopharynx, which we propose to be likely due to the sequestration of these cells within these organs. The containment of these cells within the upper respiratory tract would likely prove advantageous in promoting more efficient immune responses by increasing the likelihood for antigenic encounter and consequently facilitating more rapid, localised responses. Results from a previous study had indicated higher anti-pneumococcal responses by tonsil compared to blood CD4 T cells but failed to determine whether such a difference was significant [13]. Herein, we show that this is indeed the case.

Assessment of the pneumococcal-specific responses by mucosal CD4 T cells with age revealed a gradual age-related increase in the magnitude of cellular proliferation from the youngest age assessed at 2 years old until the age of approximately 20 years. Cytokine analysis revealed Th1 and Th17 type anti-pneumococcal CD4 T cell responses were most evident, suggesting the recruitment and activation of macrophage and neutrophils as a primary inductive mechanism for protection by CD4 T cells from pneumococcal carriage and disease, as observed in mouse models [11], [14]. We hypothesise that the progressive rise in CD4 T cell responses with age may be at least partially responsible for the observed decline in pneumococcal disease rates through childhood and early adulthood. Support for this proposal comes from epidemiological investigations into CAP. Epidemiological studies of CAP cases in Finland and earlier in the US found the incidence of the disease to show greatest decline during the first 14 years of life [39], [40]. This was also supported by a more recent investigation in the UK, which reported incidence rates dropping from 20 cases/10000 inhabitants to 6 cases/10000 inhabitants per year (Figure 2b). The incidence of CAP dropped more slowly by a further 15% between the ages of 15 to 29 years [21]. Thus, an inverse relationship between CAP and CD4 T cell responses can be observed, with the graph for the rates of CAP appearing to be almost a mirror image of the graph for the rate of CD4^+^ T cell proliferative responses to pneumococcus with increasing age. While this is suggestive of a role for mucosal CD4^+^ T cell responses in reducing CAP incidence, a role for other factors cannot be dismissed. Furthermore, whether the role of the CD4^+^ T cell response is mediated directly or via help for antibody production is unclear. Data from studies conducted by Laine *et al* on Kenyan subjects revealed increases in IgG and IgA to Ply as well as pneumococcal surface protein A (PspA) up to the age of 3 to 5 years which then plateau at least until 20 years [41]. These findings would indicate a contributory role for the antibody response in reducing pneumococcal disease early on in life but less so after 5 years of age when such responses plateau despite CAP levels still continuing to decline until teenage years. Thus, from the age of six years it is tempting to speculate that the CD4 T cell response is perhaps of greater relevance as antibody responses are seemingly static despite disease rates continuing to drop. Additional analysis of antibody responses, and in particular detailed characterisation of the levels and functional properties of both systemic and mucosal responses would be helpful in understanding the relative roles. Murine and studies in human childhood have shown an important role of CD4 T cells, particularly Th17 cells in inhibiting pneumococcal carriage lending support to a correlation between the cell-mediated response to pneumococcus and carriage rates of the bacteria [12]–[14]. Despite decreasing disease and carriage rates, CD4 T cell responses were observed to increase with age, we propose that multiple exposure events to *S. pneumoniae* takes place throughout life in order to maintain and, for the time period assessed in this study progressively augment the anti-pneumococcal CD4 T cell responses with age.

Mucosal T cell immune responses are commonly susceptible to the suppressive actions of Treg cells, and herein for the first time, we observed this to be also the case with adult human anti-pneumococcal CD4 T cell responses. Interestingly a significant Treg effect was observed during the ages when relatively strong anti-pneumococcal CD4 T cell responses have developed. Such regulation may be useful at this stage of life when these more robust responses may have a greater likelihood to cause bystander damage to host tissues following an immune response to pneumococci. Furthermore, strong responses to colonizing pneumococcus may lead to the damage of mucosal cell walls and permit bacterial penetration into tissues resulting in infection and disease. Indeed, recent studies in mice have observed the ability of gut commensal bacteria to induce the generation of Treg cells within the mucosal tissue in order to inhibit inflammatory responses that can cause immunopathology and lead to autoimmune disease [42], [43]. With the use of novel pneumococcal specific tetramers bound to epitopes of Ply, we were able to confirm the presence of Treg cells specific for pneumococcal Ply in tonsillar populations. It is therefore likely that these cells, as well as Treg which may recognise and respond to other pneumococcal antigens, are involved in the observed suppression of CD4 T cell responses to pneumococcus. Within mucosal lymphoid tissue we observed that at least 1 per approximately 200 FoxP3^+^/CD127^low/-^ CD4^+^ Treg cells in the tonsil were specific for Ply. This high incidence may reflect a level of specific induction at, or retention of anti-pneumococcal Treg cells within this site. Importantly the frequency of such cells in the peripheral blood was much lower. In future studies the novel Ply presenting tetramers that we have generated would be invaluable tools in enabling a comparison of the anti-Ply CD4 T cells in each compartment.

The reason for the delay in the development of Treg responses with age is unclear. We hypothesise that this may be a consequence of the types of encounter that take place between the host and the pneumococcus. It is possible that, during childhood, the greater propensity for colonisation events to become associated with infection favours the development of responses lacking T cell regulation. However, as immune responses increase with age, infection becomes rarer and the balance shifts toward carriage without invasion, which might subsequently predispose to the development of regulation. Alternatively, this observation may be due to differing carriage rates of the bacteria during aging. Support for this comes from our previous study assessing the CD4 T cell response to *N. meningitidis* (MenB) which like that of *S. pneumoniae* also became subject to Treg cell suppression [20]. Unlike in *S. pneumoniae* however, Treg suppression of MenB responses became evident earlier on in life between 8–11 years of age. While carriage rates are highest for *S. pneumoniae* during approximately the first decade of life and dropping to just below 10% by mid-teens onwards, for MenB carriage does not reach peak levels until late teens with pre-teens prevalence rates remaining below 10% [44], [45]. Thus, with regulation for the two bacteria appearing at ages when carriage rates for both are also similar (i.e. at just below 10%), it is tempting to speculate for the role of carriage in the development of a regulatory mechanism. A recent study by Zhang *et al* suggested that the adenoids of young children who were colonised, but not those who were not colonised with *S. pneumoniae*, contained Treg that could suppress anti-pneumococcal T cell responses [46]. Although they were not able to confirm that the Treg that they studied were pneumococcus specific as opposed to being activated polyclonally to factors such as TLR ligands in the preparations or identify the mechanisms utilised by the Tregs for suppression, their findings do support a possible role for colonization in the development of regulation. We did not distinguish between carriers and non-carriers in our studies and accordingly did not see evidence of Treg controlling reactivity in such young children. While Zhang *et al's* findings suggests a direct correlation between colonisation and Treg suppression of anti-pneumococcal CD4 T cells in infants, the presence of Tregs despite the observed low levels of colonisation in adults [12], [14] would indicate that pneumococcal-specific Treg become a stable part of the repertoire with age. Transient Treg responses in children may promote colonisation and therefore be undesirable. In adults, where colonisation is less frequent, they may have a different role altogether; to dampen potentially pathological immune responses during pneumococcal exposure and promote a beneficial profile of immunity at the mucosa.

Although the case for the majority, not all subjects above the age of 16 years displayed Treg inhibition of their anti-pneumococcal CD4 T cell immune responses, with some even showing decreases in responses following depletion of CD25^hi^ cells. Such variation is almost inevitable in complex human systems, and may possibly be due to differences in bacterial carriage and/or differences in the number of *S. pneumoniae* exposures throughout life, as well as other factors affecting the state of the local immune system at the time of the study. For example, in individuals with an ongoing mucosal response effector cells as well as Treg cells may be depleted using CD25, and this could deplete the proliferating pool as well as those potentially capable of controlling proliferation.

The Treg cells observed in our study mediated suppression using the inhibitory cell surface molecules CTLA-4 and PDL-1 in this process. CTLA-4 on Treg cells may inhibit pneumococcal specific responses by preventing and downregulating cell co-stimulation via B7.1/B7.2 and CD28 interactions between antigen presenting cells and effector T cells respectively [33], [47]. Treg cells may utilise PDL-1 in their suppression by engaging programmed death 1 (PD1) and/or B7.1 on effector T cells to attenuate T cell receptor (TCR) signalling, partly by upregulating the T cell inhibitory basic leucine transcription factor (BATF) in order to suppress T cell activation and cytokine production [48], [49]. It is interesting to note that both CTLA-4 and PDL-1 can bind B7.1 in light of a previous study observing the capacity for Treg cells to induce their inhibition through engagement of B7 molecules expressed on their target T cells [50].

The role of the antibody response in host defence against pneumococcal disease has been extensively documented, and it is therefore interesting to consider whether the Treg characterised here may affect these responses directly or indirectly via their control of effector T cells. Interestingly, studies in mice and human have indicated a capacity of Tregs to affect antibody production both *in vitro* and in mice *in vivo*. In mice increases in IgG and IgA in mucosal tissues and mucosal secretions have been observed as well as decreases in splenic IgG and IgM in the presence of Tregs [51], [52]. While in humans, Tregs were observed to inhibit IgG, IgM, IgE and IgA production by tonsillar B cells following their polyclonal activation [53]. Thus a possible additional effect of the pneumococcal specific Tregs that we have observed in this study on B cell responses to *S. pneumoniae* along with the CD4 T cell response merits assessment.

Several mouse studies have shown the importance of the Th17 CD4 response in inducing neutrophil and monocytes/macrophage mediated clearance of colonizing bacteria. Studies by Lu *et al* and Mureithi *et al* have observed *in vitro* IL-17 production following challenge with pneumococcal antigens with Lu *et al* showing an enhancing effect of IL-17 on the phagocytic killing of pneumococci by human neutrophil cells [14], [15]. Our assessment of cytokine production by adult mucosal CD4 T cells post-pneumococcal antigen challenge also revealed increases in IL-17 levels. Such Th17 responses may limit colonization in this age group and since colonization is a prerequisite of disease, may consequently contribute to the lower disease rates observed in human adults [4], [45]. However, our data additionally revealed that Tregs were able to significantly decrease the observed IL-17 production by anti-pneumococcal CD4 T cells, which have not yet been shown previously. The ability of Treg cells to preferentially block not only IL-17 but also IFN-γ pro-inflammatory cytokine production is interesting as this may reveal a role for these cells in blocking unwanted inflammation induced pathology at the mucosa upon contact with colonizing *S. pneumoniae*. Coincidently, neutrophils and macrophages, the main protagonist cell populations involved in protecting against pneumococcal carriage and/or disease are found to be principally mediated by these two particular cytokines [14], [15], thus providing a possible motive for the preferential effect of Tregs on their production. Other previous studies have shown the capacity for Tregs to inhibit Th17 cell responses as we have observed herein [54], [55]. It is possible that the Treg responses observed serve to moderate potentially pathological immunity at the mucosa in order to maintain a balance between the need to contend with a potentially harmful pathogen and to preserve physiological and barrier function. Whilst regulation may be evolutionary advantageous in the context of commensalism, the potential to suppress protective immunity during pneumococcal invasion may conversely facilitate disease.

Our findings have clear implications for the development of vaccines against the pneumococcus. As we move from approaches targeted solely at stimulating antibody responses to polysaccharide antigens and toward the generation of protein based pneumococcal vaccines, it will be important to consider the extent to which new approaches mimic natural immunity in providing local protection at the mucosa. Further, it will be important to recognise that vaccines that drive potent Th1 and/or Th17 responses without inducing the balancing effects of Treg could potentially lead to enhanced pathological outcomes in disease, and even during colonisation. The challenge in vaccinating adults will be to enhance the pre-existing responses that we have described as immunity wanes in the elderly, while maintaining the fine balances that mediate protection. As may be the case in other infectious diseases, vaccines should not necessarily be produced with the sole aim of stimulating as strong an immune response as is possible, but should rather be targeted at modulating immunity to achieve the desired outcome.

## Materials and Methods

### Ethics statement

Palatine tonsils and blood samples were obtained from otherwise healthy individuals (aged 2 to 39 years) undergoing routine tonsillectomy for recurrent tonsillitis or upper airway obstruction at Bristol Royal Hospital for Children, Southmead Hospital or Saint Michael's Hospital in Bristol, United Kingdom. Tonsils were collected into HBSS media (Invitrogen) supplemented with 100 U/ml penicillin, 100 µg/ml streptomycin (Sigma) and blood samples into citrate phosphate dextrose solution (Sigma). Patients with immunodeficiency or serious infections were excluded from the study. Participants with inflamed tonsils at the time of surgery were not recruited. No participants had received pneumococcal vaccine. The study was approved by, and sample collection and research were undertaken in accordance with the guidelines set out by the South Bristol local research ethics committee (reference number; E4388). Written informed consent was obtained from all participants and/or their legal guardians.

### Antigens

Pneumococcal cell culture supernatants (SPNT) from a standard encapsulated type 2 (D39) *S. pneumoniae* strain (National Collection of Type Cultures, NCTC #7466) was prepared as described previously [56] and used at a concentration of 2 µg/ml for cell stimulations; the optimal level as determined in dose response studies (data not shown). Recombinant Ply, a Ply protein with a Trp433-Phe mutation that reduces its haemolytic activity without affecting antigenicity generated as previously described [19], was added at a concentration of 0.1 µg/ml for cell stimulations. Pneumococcal antigens were tested for the presence of contaminating Gram-negative endotoxin using the colorimetric LAL assay (KQCL-BioWhittaker, Lonza). All purified proteins had endotoxin levels at concentrations that were too low to have any notable effect on CD4 T cell responses (<0.6 units per microgram of protein). Fluzone 2002–2003 formula inactivated split-virion influenza (flu) vaccine (Sanofi-Pasteur MSD) was used at 0.09 µg/ml hemagglutinin.

### Mononuclear cell isolation and CD25^hi^ cell depletion

Blood and tonsil tissue (MNC) were isolated by histopaque density gradient separation as described previously [20]. CD25^hi^ cells were depleted from ∼0.5–1×10^8^ MNC using anti-human CD25 coated MACS microbeads (Miltenyi Biotec) and magnetic cell sorting (MACS) on LD columns (Miltenyi Biotec) according to the manufacturer's instructions. Purity of depleted cells were typically >96% as assessed by flow cytometry using APC labelled anti-human CD25 antibody (BD Pharmingen). Approximately 8–10% of tonsil lymphocytes were found to be CD25^hi^.

Cells were resuspended at 0.8×10^6^/ml in RPMI 1640 media containing 2% human serum (Sigma), 2 mmol L-glutamine (Sigma), 100 U/ml penicillin (Sigma) and 100 µg/ml streptomycin (Sigma). Cells were plated at 1 ml/well in 48 well plates (Corning) and stimulated with flu, pneumococcal supernatant or Todd Hewitt Broth (negative control), Ply or left unstimulated as negative control for up to 9 days, at 37°C/5%CO_2_. In some stimulation experiments the cell culture supernatants were collected 5 days post stimulation and stored at −70°C for subsequent cytokine assessment. Cells were subsequently analysed for cell proliferation.

For the CD25^hi^ add back experiments, MACS purified CD25^hi^ cells were added to 0.8×10^6^ CD25^hi^ depleted cells per ml media at the original (8×10^4^ i.e. 10%) or 3 times the original (i.e. 2.4×10^5^) CD25^hi^ cell proportion in undepleted MNC.

### CD4+ T cell proliferation assay

Prior to stimulation, cells were labelled with CFSE dye (Invitrogen) according to the manufacturer's instructions, in order to permit flow cytometric tracking of cell division. Cells were stained with anti-human CD4- phycoerythrin (PE)-Cy7 antibody (BD Pharmingen) for 30 mins at 4°C and then with the vital dye TOPRO3 (Invitrogen), according to the manufacturer's instructions just prior to flow cytometric (FACS) analysis. Cells were analysed using FACS Canto (Becton Dickinson) acquiring 20000 lymphocytes (gated according to forward and side scatters). FACS results were subsequently analysed with FlowJo (TreeStar). The identification of cells that have undergone cellular division and the gating of FACS dot plots according to CFSE staining was performed as described previously [13] and consequently background (i.e. media alone for Ply stimulated or Todd-Hewitt broth treated for SPNT stimulated) proliferation were subtracted from values of stimulated cells. Dead cells (i.e. cells stained with TOPRO3) were excluded from the flow cytometric analysis. Our studies have shown that the data obtained with this technique, which allows identification of the dividing cells, directly correlates with that obtained from ^3^H-thymidine incorporation experiments from matched samples (data not shown).

### Cytokine measurement

IL- 2, 5, 10, 17, TNF-α and IFN-γ levels in the cell culture supernatants were quantified 7 days post stimulation with Ply by Luminex xMAP technology for cytokine quantification and Luminex 200 exponent system (Luminex) according to the manufacturer's instructions. Additionally IL-10, -17, TNF-α and IFN-γ production by CD4^+^ T cells was measured by intracellular cytokine flow cytometric analysis. Briefly, at day 6 to 7 of culture, cells were restimulated for 12 hours with Ply antigen and BD Golgi Stop protein transport inhibitor (BD Bioscience) was added for the last 8 hours. Cells were stained with Live/Dead near IR Stain kit (Invitrogen) according to the manufacturer's instructions, in order to exclude dead cells from flow cytometric analysis and then with anti-human CD4-V450 (BD Pharmingen). Cells were fixed and permeabilized using BD cytofix/cytoperm kit (BD Biosciences) according to manufacturer's instructions and were subsequently stained with antibodies to TNFα-alexa flour 700 (BD Pharmingen) and IL-10-APC (BD Pharmingen) or IL-17- alexa flour 700 (BD Pharmingen) and IFNγ-APC (BD Pharmingen) at 4°C for 30 mins and analysed with the BD LSR flow cytometer (Becton Dickinson). The percentage of cells positively staining for cytokines in unstimulated samples were subtracted from the percentage of positively cytokine stained cells in the stimulated samples.

### CTLA-4 and PDL-1 blocking

For pre-blocking experiment, 5×10^6^ CD25^hi^ CFSE stained cells obtained during CD25^hi^ depletion by magnetic cell sorting on LD columns as mentioned above in 1 ml RPMI 1640 media containing 1% human serum, 2 mmol L-glutamine, 100 U/ml penicillin and 100 µg/ml streptomycin were treated with 5 µg/ml anti-human CTLA-4 (eBoscience) or 15 µg/ml anti-human PDL-1 (eBoscience) or appropriate isotype-matched antibody controls (eBoscience) and incubated for 3 hours at 37°C/5%CO_2_. Cells were extensively washed and added back to CFSE stained CD25^hi^ depleted cell fraction at a ratio of 1∶10. Flow cytometric analysis revealed >80% of the CD25^hi^ cells to be successfully blocked. Cells were stimulated with antigens and left for up to 8 days in culture at 37°C/5%CO_2_ and then analysed for cellular proliferation by CFSE staining.

### Generation of tetramers

Biotinylated HLADR0401 MHCII tetramers bound to streptavidin-PE were generated at the Benaroya Research Institute (Seattle, USA). Four different Ply 13mer peptide epitopes (P1; SerAspIleSerValThrAlaThrAsnAspSerArgLeu, P2; ArgProLeuValTyrIleSerSerValAlaTyrGlyArg, P3;ValTyrLeuLysLeuGluThrThrSerLysSerAspGlu, P4; ThrSerPheLeuArgAspAsnValValAlaThrPheGln) were identified from the complete Ply protein amino acid sequence by using TEPITOPE (Vaccinome) [57]. Each Ply epitope was tested for its capacity to induce proliferation of Ply-specific T cell lines in order to determine the potential applicability of the epitopes for detecting CD4 T cells. Tetramers were generated by loading the Ply peptides onto empty biotinylated DR0401 molecules and subsequent cross-linking with streptavidin-PE [58]. To test whether each tetramer were able to bind Ply specific tonsil CD4 T cells, anti-Ply T cell lines were generated from blood CD4+ T cells of HLA DRB1^*^04 expressing individuals. Briefly, fresh PBMC were depleted of CD8^+^ cells by magnetic cell sorting using anti-CD8 magnetic beads (Miltenyi) according to the manufacturer's guide and stimulated with 0.2 µg/ml Ply peptide in RPMI 1640 medium containing 10% human serum. The plates were incubated at 37°C in 5% CO_2_, and after 7 days, the medium was replaced and 25 U/ml IL-2 (Peprotech) added. Cells were restimulated with peptide, IL-2 and irradiated autologous PBMC every 12–14 days. Cell lines in 1% human serum RPMI were stained with a tetramer presenting one of the four Ply epitopes at 10 µg/ml for 1 hour at 37°C, 5% CO_2_. Subsequent FACS analysis revealed tetramers presenting P1, P3 and P4 Ply epitopes were bound by at least 6% of cells while P2 were bound by <1% and used as a negative control.

### Tetramer staining

CD4+CD25 enriched tonsillar MNC or PBMC from HLADR0401 expressing subjects above 20 years old were obtained by magnetic sorting using the CD4 CD25 enrichment kit (Miltenyi Biotec) according to the manufacturers instruction (typically >80% of cells were CD4^+^ CD25^+^). Enriched cells were stained with either streptavidin-PE (Becton Dickenson) as a negative control, P1, P3 and P4 tetramers at 10 µg/ml each or 30 µg/ml of P2 tetramer as an additional negative control as previously. Cells were stained with anti-human CD4-PECy7 (Becton Dickenson), anti-human CD127-FITC (eBioscience) according to manufacturer's instruction followed by intracellular staining with anti-human FoxP3-APC (eBioscience) using FoxP3 staining buffer set (eBioscience) according to the manufacturer instructions and subsequently analysed by FACS and FlowJo.

### Statistical analysis

Distribution of the data was determined using the Kolmogorov-Smirnov test. When data was found to be normally distributed, differences between two groups were tested using paired or unpaired students *t-* test accordingly. The Wilcoxon signed rank test was used to test differences between undepleted MNC and CD25 depleted groups following antigenic stimulation by SPSS statistical analysis software (IBM). Two way ANOVA was used for comparing results of untreated, flu, Ply and SPNT treated groups and for the effect of Treg study and for comparing undepleted MNC, CD25-depleted, IgG blocked and CTLA-4 or PDL-1 blocked groups in the CTLA-4 or PDL-1 blocking study by SPSS statistical analysis software.

### Accession numbers

Amino acid sequence for pneumolysin was obtained from GenBank; Accession:ADF28490 (DBSOURCE - GU968411.1). GI: 294652455.

## References

[1] (1998). WHO meeting on maternal and neonatal pneumococcal immunization.. Wkly Epidemiol Rec.

[2] Mulholland EK, Adegbola RA (2005). Bacterial infections—a major cause of death among children in Africa.. N Eng J Med.

[3] Rudan I, Tomaskovic L, Boschi-Pinto C, Campbell H (2004). on behalf of WHO Child Health Epidemiology Reference Group. Global estimate of the incidence of clinical pneumonia among children under five years of age.. Bull World Health Organ.

[4] Hussain M, Melegaro A, Pebody RG, George R, Edmunds WJ (2005). A longitudinal household study of Streptococcus pneumoniae nasopharyngeal carriage in a UK setting.. Epidemiol Infect.

[5] Obaro SK, Adegbola RA, Banya WA, Greenwood BM (1996). Carriage of pneumococci after pneumococcal vaccination.. Lancet.

[6] Lipsitch M, Whitney CG, Zell E, Kaijalainen T, Dagan R (2005). Are anticapsular antibodies the primary mechanism of protection against invasive pneumococcal disease?. PLoS Med.

[7] Robinson KA, Baughman W, Rothrock G, Barrett NL, Pass M (2001). Epidemiology of invasive *Streptococcus pneumoniae* infections in the United States, 1995–1998: Opportunities for prevention in the conjugate vaccine era.. JAMA.

[8] Hill PC, Akisanya A, Sankareh K, Cheung YB, Saaka M (2006). Nasopharyngeal carriage of *Streptococcus pneumoniae* in Gambian villagers.. Clin Infect Dis.

[9] Musher DM, Groover JE, Rowland JM, Watson DA, Struewing JB (1993). Antibody to capsular polysaccharides of *Streptococcus pneumoniae* Prevalence, persistence, and response to revaccination.. Clin Infect Dis.

[10] Rapola S, Jäntti V, Haikala R, Syrjänen R, Carlone GM (2000). Natural Development of Antibodies to Pneumococcal Surface Protein A, Pneumococcal Surface Adhesin A, and Pneumolysin in Relation to Pneumococcal Carriage and Acute Otitis Media.. J Infect Dis.

[11] Zhang Z, Clarke TB, Weiser JN (2009). Cellular effectors mediating Th17-dependent clearance of pneumococcal colonization in mice.. J Clin Invest.

[12] Malley R, Trzcinski K, Srivastava A, Thompson C M, Anderson P W (2005). CD4+ T cells mediate antibody-independent acquired immunity to pneumococcal colonization.. Proc Natl Acad Sci U S A.

[13] Zhang Q, Bagrade L, Bernatoniene J, Clarke E, Paton JC (2007). Low CD4 T cell immunity to pneumolysin is associated with nasopharyngeal carriage of pneumococci in children.. J Infect Dis.

[14] Lu YJ, Gross J, Bogaert D, Finn A, Bagrade L (2008). Interleukin-17A mediates acquired immunity to pneumococcal colonization.. PLoS Pathog.

[15] Mureithi MW, Finn A, Ota MO, Zhang Q, Davenport V (2009). T cell memory response to pneumococcal protein antigens in an area of high pneumococcal carriage and disease.. J Infect Dis.

[16] Malley R (2010). Antibody and cell-mediated immunity to *Streptococcus pneumoniae*: implications for vaccine development.. J Mol Med.

[17] van Kempen MJ, Rijkers GT, Van Cauwenberge PB (2000). The immune response in adenoids and tonsils.. Int Arch Allergy Immunol.

[18] Austrian R (1986). Some aspects of the pneumococcal carrier state.. J Antimicrob Chemother.

[19] Paton JC, Lock RA, Lee CJ, Li JP, Berry AM (1991). Purification and immunogenicity of genetically obtained pneumolysin toxoids and their conjugation to *Streptococcus pneumoniae* type 19F polysaccharide.. Infect Immun.

[20] Davenport V, Groves E, Hobbs CG, Williams NA, Heyderman RS (2007). Regulation of Th-1 T cell-dominated immunity to *Neisseria meningitidis* within the human mucosa.. Cell Microbiol.

[21] Myles PR, McKeever TM, Pogson Z, Smith CJ, Hubbard RB (2009). The incidence of pneumonia using data from a computerized general practice database.. Epidemiol Infect.

[22] Oldenhove G, de Heusch M, Urbain-Vansanten G, Urbain J, Maliszewski C (2003). CD4+ CD25+ regulatory T cells control T helper cell type 1 responses to foreign antigens induced by mature dendritic cells in vivo.. J Exp Med.

[23] Hori S, Carvalho TL, Demengeot J (2002). CD25+CD4+ regulatory T cells suppress CD4+ T cell-mediated pulmonary hyperinflammation driven by *Pneumocystis carinii* in immunodeficient mice.. Eur J Immunol.

[24] Zhang X, Izikson L, Liu L, Weiner HL (2001). Activation of CD25(+)CD4(+) regulatory T cells by oral antigen administration.. J Immunol.

[25] Sakaguchi S, Sakaguchi N, Asano M, Itoh M, Toda M (1995). Immunologic self-tolerance maintained by activated T cells expressing IL-2 receptor alpha-chains (CD25). Breakdown of a single mechanism of self-tolerance causes various autoimmune diseases.. J Immunol.

[26] Hara M, Kingsley CI, Niimi M, Read S, Turvey SE (2001). IL-10 is required for regulatory T cells to mediate tolerance to alloantigens *in vivo*.. J Immunol.

[27] O'Mahony C, Scully P, O'Mahony D, Murphy S, O'Brien F (2008). Commensal-Induced Regulatory T Cells Mediate Protection against Pathogen-Stimulated NF-κB Activation.. PLoS Pathogens.

[28] Stoop JN, van der Molen RG, Baan CC, van der Laan LJ, Kuipers EJ (2005). Regulatory T cells contribute to the impaired immune response in patients with chronic hepatitis B virus infection.. Hepatology.

[29] Weiss L, Donkova-Petrini V, Caccavelli L, Balbo M, Carbonneil C (2004). Human immunodeficiency virus-driven expansion of CD4+CD25+ regulatory T cells, which suppress HIV-specific CD4 T-cell responses in HIV-infected patients.. Blood.

[30] Faria AM, Weiner HL (2005). Oral tolerance.. Immunol Rev.

[31] Sakaguchi S, Miyara M, Costantino CM, Hafler DA (2010). FOXP3^+^ regulatory T cells in the human immune system.. Nat Revs Immunol.

[32] Strauss L, Bergmann C, Szczepanski M, Gooding W, Johnson JT (2007). A unique subset of CD4^+^CD25^high^Foxp3^+^+ T cells secreting interleukin-10 and transforming growth factor-β1 mediates suppression in the tumor microenvironment.. Clin Cancer Res.

[33] Takahashi T, Tagami T, Yamazaki S, Uede T, Shimizu J (2000). Immunologic self-tolerance maintained by CD25^+^CD4^+^ regulatory T cells constitutively expressing cytotoxic T lymphocyte-associated antigen 4.. J Exp Med.

[34] Read S, Greenwald R, Izcue A, Robinson N, Mandelbrot D (2006). Blockade of CTLA-4 on CD4+CD25+ regulatory T cells abrogates their function *in vivo*.. J Immunol.

[35] Belkaid Y, Blan RB, Suffi I (2006). Natural regulatory T cells and parasites: a common quest for host homeostasis.. Immunol Rev.

[36] Sandner SE, Clarkson MR, Salama AD, Sanchez-Fueyo A, Domenig C (2005). Role of the programmed death-1 pathway in regulation of alloimmune responses in vivo.. J Immunol.

[37] Wong RM, Scotland RR, Lau RL, Wang C, Korman AJ (2007). Programmed death-1 blockade enhances expansion and functional capacity of human melanoma antigen-specific CTLs.. Int Immunol.

[38] Laughlin EM, Miller JD, James E, Fillos D, Ibegbu CC (2007). Antigen-Specific CD4+ T Cells Recognize Epitopes of Protective Antigen following Vaccination with an Anthrax Vaccine.. Infect Immun.

[39] Jokinen C, Heiskanen L, Juvonen H, Kallinen S, Karkola K (1993). Incidence of community-acquired pneumonia in the population of four municipalities in Eastern Finland.. Am J Epidemiol.

[40] Foy HM, Conney MK, Allan I (1979). Rates of pneumonia during influenza epidemics in Seattle, 1964 to 1975.. J Am Med Assoc.

[41] Laine C, Mwangi T, Thompson CM, Obiero J, Lipsitch M (2004). Age-specific immunoglobulin g (IgG) and IgA to pneumococcal protein antigens in a population in coastal Kenya.. Infect Immun.

[42] Round JL, Mazmanian SK (2010). Inducible Foxp3^+^ regulatory T-cell development by a commensal bacterium of the intestinal microbiota.. Proc Natl Acad Sci U S A.

[43] Livingston M, Loach D, Wilson M, Tannock GW, Baird M (2010). Gut commensal Lactobacillus reuteri 100-23 stimulates an immunoregulatory response to L. reuteri.. Immunol Cell Biol.

[44] Christensen H, May M, Bowen L, Hickman M, Trotter CL (2010). Meningococcal carriage by age: a systematic review and meta-analysis.. Lancet Infect Dis.

[45] Bogaert D, van Belkum A, Sluijter M, Luijendijk A, de Groot R (2004). Colonisation by *Streptococcus pneumoniae* and *Staphylococcus aureus* in healthy children.. The Lancet.

[46] Zhang Q, Leong SC, McNamara PS, Mubarak A, Malley R (2011). Characterisation of Regulatory T Cells in Nasal Associated Lymphoid Tissue in Children: Relationships with Pneumococcal Colonization.. PLoS Pathog.

[47] Wing K, Onishi Y, Prieto-Martin P, Yamaguchi T, Miyara M (2008). CTLA-4 Control over Foxp3^+^ Regulatory T Cell Function.. Science.

[48] Butte MJ, Keir ME, Phamduy TB, Sharpe AH, Freeman GJ (2007). Programmed Death-1 Ligand 1 Interacts Specifically with the B7-1 Costimulatory Molecule to Inhibit T Cell Responses.. Immunity.

[49] Quigley M, Pereyra F, Nilsson B, Porichis F, Fonseca C (2010). Transcriptional analysis of HIV-specific CD8+ T cells shows that PD-1 inhibits T cell function by upregulating BATF.. Nat Med.

[50] Paust S, Lu L, McCarty N, Cantor H (2004). Engagement of B7 on effector T cells by regulatory T cells prevents autoimmune disease.. Proc Natl Acad Sci U S A.

[51] Iikuni N, Lourenço EV, Hahn BH, La Cava A (2009). Cutting edge: Regulatory T cells directly suppress B cells in systemic lupus erythematosus.. J Immunol.

[52] Cong Y, Feng T, Fujihashi K, Schoeb TR, Elson CO (2009). A dominant, coordinated T regulatory cell-IgA response to the intestinal microbiota.. Proc Natl Acad Sci U S A.

[53] Lim HW, Hillsamer P, Banham AH, Kim CH (2005). Cutting Edge: Direct Suppression of B Cells by CD4^+^CD25^+^ Regulatory T Cells.. J Immunol.

[54] Crome SQ, Clive B, Wang AY, Kang CY, Chow V (2010). Inflammatory Effects of Ex Vivo Human Th17 Cells Are Suppressed by Regulatory T Cells.. J Immunol.

[55] Reynolds AD, Stone DK, Hutter JA, Benner EJ, Mosley RL (2010). Regulatory T Cells Attenuate Th17 Cell-Mediated Nigrostriatal Dopaminergic Neurodegeneration in a Model of Parkinson’s disease.. J Immunol.

[56] Zhang Q, Bernatoniene J, Bagrade L, Pollard AJ, Mitchell TJ (2006). Serum and mucosal antibody responses to pneumococcal protein antigens in children: relationships with carriage status.. Eur J Immunol.

[57] Sturniolo T, Bono E, Ding J, Raddrizzani L, Tuereci O (1999). Generation of tissue-specific and promiscuous HLA ligand databases using DNA microarrays and virtual HLA class II matrices.. Nat Biotechnol.

[58] Novak EJ, Liu AW, Nepom GT, Kwok WK (1999). MHC class II tetramers identify peptide-specific human CD4^+^ T cells proliferating in response to influenza A antigen.. J Clin Invest.

